# Average Daily Gain and Energy and Nitrogen Requirements of 4-Month-Old Female Yak Calves

**DOI:** 10.3389/fvets.2022.906440

**Published:** 2022-07-13

**Authors:** Binqiang Bai, A. Allan Degen, Xiaodong Han, Lizhuang Hao, Yayu Huang, Jianzhang Niu, Shujie Liu

**Affiliations:** ^1^Key Laboratory of Plateau Grazing Animal Nutrition and Feed Science of Qinghai Province, State Key Laboratory of Plateau Ecology and Agriculture, The Academy of Animal and Veterinary Sciences, Qinghai University, Xining, China; ^2^Desert Animal Adaptations and Husbandry, Wyler Department of Dryland Agriculture, Blaustein Institutes for Desert Research, Ben-Gurion University of the Negev, Beer Sheva, Israel; ^3^GenPhySE, Université de Toulouse, Institut Nationale de la Recherche Agronomigue, Institut National Polytechnique de Toulouse, École Nationale Vétérinaire de Toulouse, Castanet Tolosan, France

**Keywords:** milk intake, efficiency of utilization of energy for maintenance, efficiency of utilization of energy for growth, biological value of N, composition of retained energy

## Abstract

There is little information available on milk intake and energy and nitrogen requirements of growing yak calves. This study aimed to fill this important gap, as this information could be beneficial in designing a system to wean yak calves earlier than in natural time. We determined the average daily gain and energy and nitrogen balances and requirements of 4-month-old female yak calves (48.8 ± 2.45 kg, *n* = 8). The calves were allowed to suck once a day and were fed an *ad libitum* concentrate: hay diet at a ratio of 60:40. Milk intake averaged 540 ± 26 g/d, yielding 2.28 ± 0.112 MJ/d, which was 13% of the gross energy intake (GEI). The digestible energy intake (DEI):GEI ratio was 0.681, metabolizable energy intake (MEI):DEI was 0.913, and MEI:GEI was 0.621. The average daily gain of the calves was 433 ± 153.1 g/d, which consisted of 78.0 ± 8.99 g protein, 52.7 ± 23.74 g fat, and 302.3 ± 95.1 g water, that is, 18.0% protein, 13.0% fat and 69.8% water. There were 130.7 g of body solids and 9.06 MJ of energy in every kg of body mass gain. Of the MEI, 25.17 kJ were required for 1 g of body mass, 83.40 kJ for 1 g of body solids, and 2.62 kJ for 1 kJ of retained energy (RE), and RE was 36.6% of MEI. The maintenance energy requirement was 5.35 MJ/d, the efficiency of utilization of energy for growth (k_g_) was 0.72, and the heat increment of feeding for growth was 0.28 (1.55 MJ/d). Digestible nitrogen (N) was 0.685 while retained N (RN) was 0.489 of N intake. The N requirement for maintenance was 11.73 g/d or 0.61 g N/kg^0.75^ per day, while the biological value (BV) of N was 91.1%. The energy and N requirements for yak calves were relatively low, which could be explained, at least in part, by the high efficiency of utilization of energy and high BV of N when compared to other livestock. These findings could be beneficial in designing early weaning systems for the many Himalayan households depending on yak production for their livelihoods.

## Introduction

Yaks *(Poephagus grunniens)* are raised on the harsh alpine and subalpine meadows of the Qinghai Tibetan plateau (QTP). The plateau is characterized by severe cold, low air oxygen content, strong ultraviolet light, and a short forage growing season (1, 2). Yaks are vital for the livelihood of the herders, providing milk, meat, fiber, and fuel (dung). They are hardy ruminants that have adapted well to the extreme conditions, both anatomically and physiologically, and can survive and even thrive under extremely harsh conditions (3, 4).

Traditionally, yak calves are weaned naturally or artificially until 1.5 to 2 years of age under extensive conditions (5). Naks (female yaks) that calved in the previous year produce milk without calving, but at a lower volume, and suck their calves (6). Continuous sucking during the winter and spring seasons (from October to May) is difficult for naks on the Tibetan plateau because only sparse forage is available and air temperatures are extremely low. Naks often lose body condition and body mass at this time, and milk yields of less than 1 L/d are common (5). The poor body condition and long sucking period increase the time between calvings in naks. Earlier weaning of the calves than the natural time, as is common in dairy calves (7), could improve the reproductive rate of naks.

Yak calves generally graze with their mothers on natural pasture until weaning. However, if milk is collected from yaks for home consumption, then calves are separated from their mothers overnight, the naks are milked in the morning, and then the calves join their mothers till evening when they are separated again. Many studies have reported on milk intake and energy and nitrogen balances of growing lambs (8) and cattle calves (9), but none has been reported on yak calves. The aim of this study is to fill this important gap, as this information could be beneficial in designing a system to wean yak calves earlier than in natural time.

## Materials and Methods

The protocol and all experimental procedures on the yak calves were approved by the Committee of Animal Use, Academy of Science and Veterinary Medicine, Qinghai University (Number: QHUA-2018-0412).

### Animals, Experimental Design, and Diet

The study used eight, 4-month-old female yak calves (initial body weight = 48.8 ± 2.45 kg) that were born in late March 2018 and had been with their mothers (158.8 ±8.55 kg) on natural pasture. The calves were separated from their mothers in early July 2018, and kept in two pens, each holding four calves, for 20 days before the start of measurements. The pens had a covered section of 30 m^2^, and a connecting open-roofed area of 30 m^2^. The closed section contained a water trough and two feed troughs. The calves were allowed to suck one time each day at 07:00 h by bringing the calf to her mother in a nearby holding pen. The calves were then returned to the original pens and offered an *ad libitum* diet of concentrate and oat hay (60:40) in separate troughs twice daily (08:00 h and 18:00 h), with water available freely. The composition and nutritional level of the diet are presented in Table 1. Following the 20-day adjustment period, the yaks were weighed and were moved in groups of four (2 from each pen) into indirect open-circuit respiration calorimeter chambers (10). After 3 days of adaptation to the chambers, feed intake was measured for seven days, and feces and urine outputs, enteric CH_4_ emission, and O_2_ consumption were measured during the final five days. The calf was brought to her mother each day, and milk intake was measured by weighing the calf on a walk-on digital scale (accuracy to 10 g; model ZHGD-III, Shanghai, China) before and after sucking, as was described for yak calves (11). Each calf was released to her mother and allowed to suck until she stopped. Milk intake was recorded only when the calf did not defecate or urinate between the initial and final weighings. There were at least three milk intake measurements that met these criteria for each calf, and each measurement took <40 min. Milk samples were collected, pooled by nak, preserved with potassium dichromate, and stored at 4°C. The samples were analyzed for concentrations of fat, protein, and lactose contents (Central Milk Testing Laboratory, Beijing, China). The energy content of the milk was calculated from its composition as: 38.11 MJ/kg fat, 24.52 MJ/kg protein, and 16.52 MJ/kg lactose (12, 13).

**Table 1:** Composition and gross energy of basal diets.

**Gross energy and composition^**1**^**	**Concentrate**	**Oat hay**
Corn (g/kg)	500.00	
Soybean oil (g/kg)	166.65	
Soybean meal (g/kg)	166.65	
Cottonseed meal (g/kg)	125.00	
Calcium carbonate (g/kg)	6.70	
Sodium chloride (NaCl, g/kg)	10.00	
Premix (g/kg)	25.00	
**Nutrient values**^**2**^ **(DM basis)**		
DM (g/kg)	925.5	947.4
GE (MJ/kg)	17.15	16.63
Crude protein (g/kg)	224.3	77.5
Neutral detergent fiber (g/kg)	330.4	581.4
Acid detergent fiber (g/kg)	152.4	313.6
Ash (g/kg)	66.7	77.7

^1^*The composition per kg of premix was: vitamin A, 1,000 KIU; vitamin D3, 250 KIU; vitamin E, 1,500 IU; biotin, 200 IU; folic acid, 10 mg; niacin, 2,500 mg; iron, 3,000 mg; copper, 2,000 mg; zinc, 6,000 mg; manganese, 4,500 mg; iodine, 100 mg; selenium, 70 mg*.

^2^*DM, dry matter; GE, gross energy*.

### Digestibility Trials

Intakes of concentrate and oat hay were measured separately by the difference between the amounts offered and orts left behind. Feces were collected using fecal bags, which were emptied two times a day, and 10% of the total was kept for analysis. Twenty ml of 10% sulfuric acid were added to each 100 g of feces to prevent loss of N. Total urine was collected using a modified urine collector for humans, and 5% of the total was kept for analysis. To prevent the loss of N as ammonia, 10% sulfuric acid was added to the urine samples to maintain the urine pH below three. The feces and urine were stored at −20°C.

Feed, orts, and feces were oven-dried at 65°C for 72 h and then ground to pass through a 0.8 mm screen for chemical analyses. Dry matter (DM) was determined by drying samples at 105°C for 3 h in a moisture extraction oven [(14): method 925.45], ash was measured by burning samples at 550°C in a muffle furnace [(14): method 990.03], and organic matter (OM) was calculated by the difference between DM and ash. Total N was determined by the Kjeldahl method and crude protein was estimated as N concentration × 6.25 [(14): method 976.06]. Neutral detergent fiber, without a heat-stable amylase and expressed inclusive of residual ash, and acid detergent fiber of the feed were determined following Van Soest et al. (15).

Gross energy of feces, feed, and urine were determined using an isothermal bomb calorimeter (KDHW-800B, Henan, China). The urine energy was determined by a modification of the method described by Nijkamp (16). In brief, filter papers with and without weighed urine were dried at 65°C for 12 h and then dried at 105°C for 1 h. The urine energy was calculated by the difference in energy of the filter paper with and without urine.

### Methane Emission Measurements

Methane emission measurements of the yak calves followed the protocol of Bai et al. (17). The yak calves were weighed, and then each calf in her metabolic cage was moved into an individual respiration chamber. The respiration chamber consisted of a control room and four single cells. Each cell had a total volume of 37.13 m^3^ (4.5 m length × 3.3 m height × 2.5 m width) and was constructed of double-layer stainless steel sides with a 100 mm polyurethane plate between the layers. To minimize stress on the calves, they were able to see each other through glass panes between the chambers. The chambers were kept closed, with slight negative pressure. The air temperature was maintained at 20°C and relative humidity at 75%, with a regime of 14 h light and 10 h dark. The air temperature and humidity were measured by electronic sensors (HygroClip S, Rotronic AG, Basserdorf, Switzerland). The CH_4_ production and oxygen (O_2_) consumption were measured using an open-circuit respiration system. Each calf was measured over five consecutive days and volumes of CH_4_ and O_2_ were calculated by multiplying the flow rate (>12 m^3^/h) times the difference in concentrations of air entering and leaving the chambers. The air from each cell was dried by an air-drying column (Drierite, Xenia, OH, USA) before measurement. The main equipment of the gas measurement system included a pre-treatment cabinet, vortex fan (RHG210-7H1, Beijing, P.R. China), mass flow meter (S420, Bach, Ochtrup, Germany), CH_4_ analyzer (QGS-08C, Sick Maihak, Waldkirch, Germany), CO_2_ analyzer (QGS-08C, Sick Maihak, Waldkirch, Germany), and O_2_ analyzer (PM710, Systech Illinois, Oxford, UK). High purity carbon dioxide (99.99%) was passed into the cabinet, and the flow rate was calibrated by checking the recovery of carbon dioxide gas (18). The recovery was 98 - 100%. Before the start of each period, CH_4_ and O_2_ of known concentrations were used to calibrate the analyzers (0-884 ppm for CH_4_; 21.46% for O_2_). Data were collected using a data logger (Kooland Company Ltd., Beijing, China), and were recorded every 25 min.

### Calculations of Energy and Nitrogen Balances

The volume of the flow meter was converted to standard temperature pressure, dry (STPD: 0°C and 760 mm Hg). The CH_4_ emission in g was converted to L using the conversion factor of 0.7143 g/L and was converted to CH_4_ energy (CH_4_-E) using the conversion factor of 55.65 kJ/g (19). Metabolizable energy (ME) intake was determined as: DE intake – (urine energy + CH_4_-E).

Equations used to calculate energy balance were as follows:

Methane energy (CH_4_-E, MJ/d) = CH_4_ (L/d) × 0.03954 MJ/L (19).

Heat production (HP, MJ/d) = O_2_ (L/d) × 0.0217 (MJ/L) (20).

Metabolizable energy intake (MEI, MJ/d) = GEI − (FE + UE + CH_4_-E) = DEI–(UE + CH_4_-E).

Retained energy (RE, MJ/d) = MEI (MJ/d) – HP (MJ/d).

Nitrogen balance was calculated as:

Digestible nitrogen (DN, g/d) = N intake (g/d) – Fecal nitrogen (FN, g/d).

Retained nitrogen (RN, g/d) = N intake (g/d) – FN (g/d) – Urinary nitrogen (UN, g/d).

The composition of body mass change was calculated as follows:

Change in body protein (CBP, g/d) = RN × 6.25

Change in body protein energy (CBPE, kJ/g) = CBP (g) × 23.43 (kJ/g)

Change in body fat energy (CBFE, kJ/g) = RE (kJ/d) – CBPE (kJ/g)

Change in body fat (g/d) = CBFE (kJ/d)/39.32 (kJ/g)

Change in body water = ADG (g) – [CBP (g) + CBF (g)]

Energy requirement for maintenance was taken at RE = 0 from the regression of RE on MEI. The regression equation took the form:

RE (MJ/d) = a MEI (MJ/d) + b

The slope of regression (a) was the efficiency of utilization of energy for growth (k_g_), as all the calves were gaining weight and 1 – k_g_ was the heat increment of feeding for growth (HIF_g_).

The requirement of N for maintenance was taken at retained N (RN) = 0 from the regression of RN on total N intake (TNI). The regression equation took the form:

RN (g/d) = a TNI (g/d) + b

Crude protein requirement for maintenance was calculated as N requirement for maintenance × 6.25.

The biological value (BV) of N, which represents the efficiency of deposition of ingested N, was calculated following Mitchell (quoted by 19) as:

BV (%) = 100 × N intake – [(fecal N – metabolic N) + (urine N – endogenous N)]/N intake – (fecal N – metabolic N); where metabolic N = 2.2 g N/kg DMI and endogenous N = 0.2 g N/kg^0.75^.

## Results

### Energy Intake and Average Daily Gain

The ratio of concentrate:hay was offered at 60:40, but the actual intake was 47:53. The composition of milk averaged 6.06% fat, 5.05% protein, and 4.75% lactose, and the gross energy averaged 4.22 MJ/kg. Milk intake averaged 540 ± 26 g/d or 2.28 ± 0.112 MJ/d, which was 8.7% of the total DMI and 13.0% of the total GEI (Table 2). The ratio of DEI:GEI was 0.681, of MEI:DEI was 0.913, and of MEI:GEI was 0.621. Total N intake averaged 25.5 ± 1.59 g/d, digestible N intake was 17.5 ± 1.61 g/d, and retained N was 12.5 ± 1.44 g/d (Table 3). Daily gain of the calves averaged 433 ± 153.1 g/d, of which 78.0 ± 8.99 g were protein, 52.7 ± 23.74 g were fat and 302.3 ± 95.1 g were water, that is, 18.0% protein, 12.2% fat and 69.8% water (Table 4). (The water included a small amount of retained ash). Therefore, every kg gained in body mass included 130.7 g of body solids and 9.06 MJ of energy. Of the 10,900 kJ MEI, 25.17 kJ were required for 1 g body mass, 83.40 kJ for 1 g of body solids, and 2.62 kJ for 1 kJ body energy. Retained energy was 36.6% of the MEI.

**Table 2:** Body weight, average daily gain, dry matter and nutrient intake, and digestibility in 4-month-old female yak calves (means ± SE; *n* = 8).

**Measurement**	**Values**
Initial body weight (kg)	48.8 ± 2.45
Final body weight (kg)	53.1 ± 2.67
ADG (g/d)	433 ± 153.1
DMI: non-milk (g/d)	904 ± 107.6
Concentrate (g/d)	423 ± 85.5
Hay (g/d)	482 ± 44.2
GEI: non-milk (MJ/d)	15.26 ± 1.830
Milk intake (g/d)	540 ± 26.7
Milk DMI (g/d)	85.9 ± 4.31
GEI: Milk (MJ/d)	2.28 ± 0.112
Total DMI (g/d)	990 ± 106.4
Total GEI (MJ/d)	17.54 ± 1.799
Fecal DM output (g/d)	342 ± 36.7
DM digestibility (g/kg)	655 ± 2.1
Fecal energy (MJ/d)	5.61 ± 0.50
DEI (MJ/d)	11.94 ± 0.121
DEI:GEI (MJ/MJ)	0.681 ± 0.031
Urine output (g/d)	665 ± 83.2
Urine energy (MJ/d)	0.69 ± 0.22
CH_4_ emission (g/d)	6.25 ± 1.43
CH_4_ Energy (MJ/d)	0.35 ± 0.080
MEI (MJ/d)	10.90 ± 0.121
MEI:DEI (MJ/MJ)	0.913 ± 0.0234
MEI:GEI (MJ/MJ)	0.621 ± 0.0144
HP (MJ/d)	6.91 ± 0.096
RE (MJ/d)	3.99 ± 1.35

**Table 3:** Nitrogen intake, output, and balance in 4-month-old female yak calves (means ± SE; *n* = 8).

**Measurement**	**Values**
Total N intake (g/d)	25.53 ± 1.593
Concentrate (g/d)	15.19 ± 1.400
Hay (g/d)	5.97 ± 0.443
Milk (g/d)	4.37 ±0.174
Total N output (g/d)	13.05 ± 0.637
Feces (g/d)	8.03 ± 0.052
Urine (g/d)	5.02 ± 0.635
Digestible N (g/d)	17.5 ± 1.61
N digestibility (g/kg)	685 ± 0.19
N retention (g/d)	12.48 ± 1.440

**Table 4:** Composition and energy content of average daily gain in 4-month-old female yak calves (means ± SE; *n* = 8).

**Measurement**	**Values**
Average daily gain (g/d)	433 ± 153.1
Protein (g/d)	78.8 ± 8.99
Fat (g/d)	56.5 ± 23.74
Water (g/d)	298.6 ± 95.1
Retained energy (MJ/d)	4.05 ± 1.35
Protein (MJ/d)	1.83 ± 0.021
Fat (MJ/d)	2.23 ± 0.936

### Metabolizable Energy and Nitrogen Requirements

**Figure 1:**
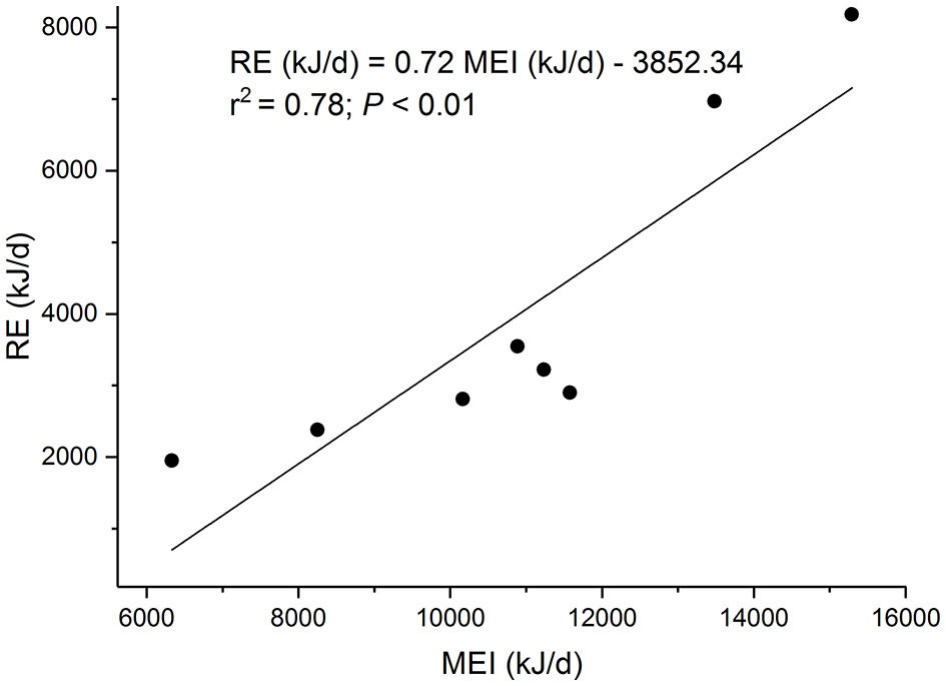
The regression of retained energy (RE) on metabolizable energy intake (MEI) in eight sucking female yak calves.

The linear regression equation of retained energy (RE, MJ/d) on metabolizable energy intake (MEI, MJ/d) took the form (Figure 1): RE = 0.72 MEI – 3.852 (r^2^ = 0.78; P < 0.01). Therefore, the maintenance energy requirement was 5.35 MJ/d, the efficiency of utilization of energy for growth (k_g_) was 0.72, and the heat increment of feeding for growth (HIF_g_) was 0.28 (1.55 MJ/d).

**Figure 2:**
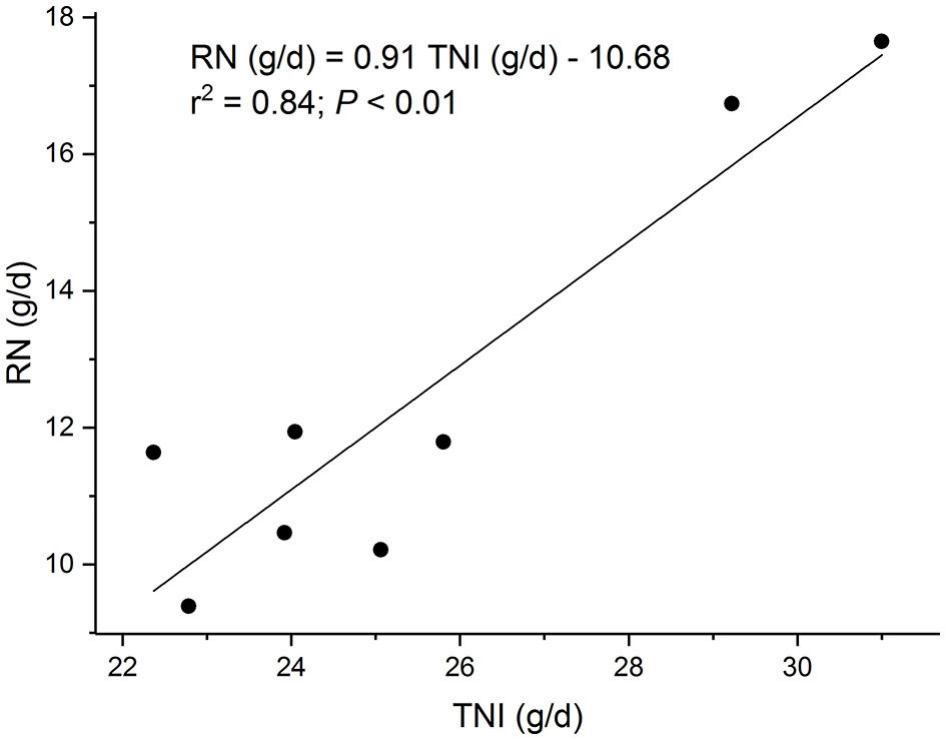
The regression of retained nitrogen (RN) on total nitrogen intake (TNI) in eight sucking female yak calves.

Apparent N digestibility was 0.685 while retained N was 0.489 of total N intake (TNI). The linear regression equation of RN (g/d) on TNI (g/d) took the form (Figure 2): RN = 0.91 TNI – 10.68 (r^2^ = 0.84; P < 0.01). Therefore, the N requirement for maintenance was 11.73 g/d or 0.61 g N/kg^0.75^ per day and the crude protein requirement was 73.3 g/d or 3.84 g CP/kg^0.75^ per day. The BV of the N was 91.1%.

## Discussion

### Average Daily Gain (ADG) and Energy Intake

Yaks are well-adapted to the harsh Himalayan highlands. However, their productivity is poor, as naks (females) calve only once every 2 to 3 years (21). The calves are small at birth, and their growth rate is slow when compared with cattle calves (22, 23). The ADG of the yak calves in this study was 433 g/d. However, comparisons with yak calves in other studies should be made with caution as ADG can vary greatly depending on such factors as management and time of year. For example, 6-month-old yak calves gained 533 to 558 g/d when naks were not milked, 457 to 482 g/d when milked once a day, and 215 to 230 g/d when milked twice a day (24). Yak calves that ran with their mothers on natural pasture, with no supplements provided to either the naks or calves, gained 213 g/d from birth to 6 months of age, and 182 g/d from 6 to 12 months of age (25). The ADG of calves sucking Maiwa naks receiving supplementary hay ranged between 300 and 316 g/d, whereas calves sucking naks not receiving supplements gained between 279 and 295 g/d from birth to 90 days of age (26). In addition, the season can influence the ADG of calves. In Tibet, in the warm season (June to September), when the calves were 3 to 4 months or 13 to 14 months of age, ADG was 180 to 250 g/d, but in the cold season (November to March), when calves were 6 to 10 months of age, calves lost between 12 and 15% of their body mass. In January, these calves lost 31 g/d (27). This would suggest that the present study was done at an optimal time for calf growth.

The milk yield of naks is relatively low, with some breeds producing <1 kg per day (22, 25). The milk intake of the yak calves in the present study was 540 g/d, and the DEI:GEI ratio for the total dietary intake was 0.681. According to NRC (28), the DE:GE ratio for milk is close to 0.97, and ratios of 0.945 (29) and 0.95 (9) have been reported for Holstein male calves consuming milk replacer. This would indicate that the concentrate and hay consumed by the yak calves in the present study had a considerably lower digestible energy. If we assume that the milk in the present study had digestible energy of 0.95, and then subtract the milk DM from the total DMI and subtract 0.05 × milk DM (g) from the total fecal output, the DE:GE ratio for the concentrate and hay combined would be 0.631. The ratios of ME:DE and ME:GE of the yak calves were also lower than the ratios reported for cattle calves consuming milk replacer. The ME:GE ratios of 0.92 (9) and 0.895 (29) were reported for Holstein calves on milk replacer, while NRC (28) suggested a ME:DE ratio of 0.96 and a ME:GE ratio of 0.93 for milk. In a study on calves, in which milk replacer was 49.6%, calf starter was 41.8% and hay was 8.6% of GEI, the DE:GE ratio was 0.802, and the ME:GE ratio was 0.751 (30), which were closer to the digestibilities in the present study. In a study on 8- to 16-month-old calves consuming a concentrate:hay diet similar to the ratio in this study, the DE:GE ratio was 0.77 (17). These latter calves were older than the calves in the present study and their rumens were better developed.

### Energy Balance and Efficiency of Utilization of Energy

We are unaware of other studies reporting milk intake and MEI of sucking yak calves and the conversion rates of MEI to body mass, total body solids gain and retained energy (RE), and the ADG:DMI ratio. Although these ratios are used frequently, they should be taken with caution when comparisons are made among studies, as they are not dependent only on the efficiency of utilization of energy intake, but also on the total MEI. Animals can increase RE only when MEI is above ME_m_, and, once reached, the RE:MEI ratio increases with an increase in MEI. In the present study, the MEI (MJ/d):RE (MJ/d) ratio was 2.73 and RE was 36.6% of MEI. In a study on beef calves gaining 706 to 993 g/d while consuming milk replacer, RE was 33.3% of MEI when MEI was 20.6 MJ/d and 35.1% of MEI when MEI was 27.5 MJ/d (31). In 2-month-old Morada Nova intact male lambs weighing 12 kg, RE was 4.9% of MEI when consuming 3.65 MJ/d, but increased to 16.6% of MEI when consuming 9.19 MJ/d (32). With the lower MEI in these lambs, most of the energy intake was allotted to maintenance and little was available for growth, but with the greater MEI, more energy was allotted to growth. Calegare et al. (31) referred to this ratio as ‘energetic efficiency'. But as mentioned above, this ratio is very dependent on intake above ME_m_. In fact, using this ratio, the energetic efficiency could be negative, which is not possible. It can be low, but not negative.

In the present study, each kg of body weight gain in the yak calves consisted of 130.7 g of body solids and 9.21 MJ. Male Holstein calves, with an initial body weight of 45 kg, were offered different amounts of milk replacer (MEI) so that the ADG ranged between 550 and 1,210 g/d. Each kg of body weight gain ranged between 264 and 408 g of body solids and 7.99 and 10.71 MJ. The RE of the yaks fell between the upper and lower values of the Holsteins, mainly because of more or less fat deposited. The ADG:DMI ratio in the yaks was 0.44. In male Holstein calves receiving different energy concentrations of milk replacer, the ADG:DMI ratio ranged between 0.51 and 0.78 (29). The digestibility of the milk replacer consumed by the Holstein calves was much greater than the digestibility of the total diet of the yak calves, and this could explain the higher ratio in the Holstein calves. Yak calves aged a year-old and weighing 90 kg gained between 330 and 411 g/d on diets of different crude protein (CP) and energy content (33). The calves consuming a low CP, low energy diet had the lowest ADG, with an ADG:DMI ratio of 0.092, whereas, calves consuming a high CP, high energy diet had the highest ADG, with an ADG:DMI ratio of 0.112. The digestibilities of the diets and the ADG were lower than the corresponding values for the yak calves in this study, which could explain the lower ADG:DMI ratios.

The MEI (MJ/d):ADG (kg/d) ratio for the yak calves in the present study was 25.2 MJ/kg. In beef calves sucking once a day and with access to corn silage *ad libitum*, the MEI:ADG ratio was 37.0 MJ/kg for males and 36.1 MJ/kg for females (31). Therefore, the MEI:ADG ratio of these beef calves was ~ 45% higher than for the yak calves. Morada Nova lambs, aged 2 months and offered diets ranging from 4.01 to 10.96 kJ/d ME, had an ADG of 16.6 to 136.0 g/d. These lambs consumed different ratios of forage:concentrate and their MEI:ADG ratios ranged between 67.6 and 210.0 MJ/kg (32). Camel calves consuming milk replacer, concentrates, and lucerne hay gained 718 g/d from 30 to 120 days of age. The average MEI of these camel calves was 18.3 MJ/d and the MEI:ADG ratio was 25.5 MJ/kg (34). The MEI:ADG ratio, referred to as a measure of ‘gross efficiency' by Calegare et al. (31), was similar between the yak and camel calves, and was generally lower than ratios reported for other species. This would suggest that yak and camel calves are “more efficient” in utilizing MEI than other species. Yaks and camels are well-adapted to extreme environments where often only sparse vegetation is available. Both the yak (35) and the camel (36) are known for their low ME_m_, which would allow more energy intake for growth and can explain, at least in part, the difference in the “gross efficiency” among the species. The composition of the ADG would also influence this ratio. Here, again, the “efficiency” could be negative, which is not possible.

The energy requirement for maintenance of the yak calves was 5.35 MJ/d or 281 kJ /kg^0.75^ per day, which was 37.1% < ME_m_ reported for yaks (35). It was also <545 kJ/kg^0.75^ per day reported for grazing yaks (23). The energetic cost of grazing could explain the higher ME_m_ in the latter study. The ME_m_ for the calves in this study included BMR and HIF_m_, and the difference between MEI and ME_m_ (10.90 – 5.35 = 5.55) included RE and HIF_g_. The RE was calculated as 3.99 MJ/d from the difference between MEI and HP. Therefore, the HIF_g_ was 2.07 MJ/d and the efficiency of utilization of energy for growth, k_g_, was 0.72 (3.99/5.55). The efficiency of utilization of energy for maintenance, k_m_, is >k_g_, therefore, the BMR for these yak calves was >3.85 (>5.35 × 0.72) MJ/d, while the heat increment of feeding for maintenance (HIF_m_) was <1.50 (<5.35 ×0.28) MJ/d. The k_g_ of 0.72 for yaks was higher than values that have been reported for cattle calves. For cattle calves, NRC (28) suggested a k_g_ of 0.69, while Diaz et al. (9) and Blome et al. (29) reported a k_g_ of 0.58 to 0.59. Overall efficiency of utilization of energy for maintenance and growth was reported as 0.69 for 1-year-old cattle calves (30) and 0.69 to 0.71 for sucking lambs (8, 37, 38). These latter studies included k_m_, which decreased the efficiency when compared to the present study. It is evident that yaks utilize energy more efficiently than other species and this could explain the relatively low ME_m_ in yaks.

### Nitrogen Balance and Biological Value of Nitrogen

Yaks are known for their low nitrogen needs. The lower requirement of N by yaks than in cattle has been attributed primarily to a more effective usage and recycling of urea (39, 40), and a greater efficiency of microbial protein synthesis (41). The Ministry of Agriculture (42) recommended a maintenance requirement of 0.88 g N/kg^0.75^ per day for cattle, which would have amounted to 17.31 g N/d for the yak calves in this study. However, the N requirement for maintenance for the yak calves was 11.73 g/d or 0.61 g N/kg^0.75^ per day, which is 32% below the amount recommended for cattle. Low maintenance N requirements, ranging between 0.40 and 0.53 g N/kg^0.75^ per day have been reported for yaks (1, 39, 43).

Nitrogen digestibility in Holstein calves consuming 19.2 to 33.9 g N/d was 85.5 to 90.2% and retained N was 39.4 to 45.9% of total N intake (29). In the present study, N digestibility was 68.5%, and retained N was 48.9% of intake energy. Therefore, N digestibly was greater in cattle than in yak calves, but the proportion of retained N was greater in yaks than in cattle. This occurred because of the lesser proportion of N lost in yak than in cattle urine, most likely due to the more efficient handling of urea.

The biological value (BV, %) of N, that is, the efficiency of deposition of ingested N, was 91.1% for the yak calves in this study. According to NRC (28), BV for milk replacer should be about 80%. Calegare et al. (31) reported a range of 57 to 70% in beef calves consuming 20.6 to 27.5 MJ MEI/d, whereas Blome et al. (29) calculated a BV of 67.3 to 71.1% for Holstein calves consuming 14.1 to 16.0 MJ MEI/d and 19.2 to 33.9 g N/d. In the latter study, there was a decrease in BV with an increase in N intake. The greater BV of N in yaks than in cattle could explain, at least in part, the lower N requirements for maintenance in yaks than in cattle.

## Conclusions

Milk intake averaged 540 g/d and daily gain averaged 433 g/d for the yak calves. Maintenance requirements for energy were 5.35 MJ/d (280 kJ/kg^0.75^ per day) and for N was 11.73 g N/d (0.061 g N/kg^0.75^ per day), while the biological value of N was 91.1%. The calves displayed a high efficiency of utilization of energy and biological value of N and low requirements of energy and N for maintenance when compared to other livestock species. Findings in this study could be beneficial in designing early weaning systems for Himalayan households dependent on yak production for their livelihoods. Results from this study are applicable for female yak calves fattened in feedlots and allowed to suck once daily. Further research is required for male yak calves under similar husbandry and for calves that have continual access to their mothers.

## Data Availability Statement

The raw data supporting the conclusions of this article will be made available by the authors, without undue reservation.

## Ethics Statement

The protocol and all procedures on the animals were approved by the Committee of Animal Use of the Academy of Science and Veterinary Medicine of Qinghai University. Written informed consent was obtained from the owners for the participation of their animals in this study.

## Author Contributions

BB and AD: formal analysis, data curation, writing—original draft preparation, and writing—review and editing. AD: supervision. XH: methodology and investigation. LH and SL: conceptualization, methodology, supervision, project administration, and funding acquisition. YH and JN: investigation. All authors contributed to the article and approved the submitted version.

## Funding

This work was funded by Special Topics of the Second Comprehensive Scientific Expedition of the Qinghai-Tibet Plateau (2019QZKK0606), Natural Science Foundation of China (NSFC, 31660673 and 32060766), the Independent Project of State Key Laboratory of Plateau Ecology and Agriculture (2019-ZZ-19), Youth Fund of Nature Fund in Qinghai Province (2022-ZJ-974Q), Key Laboratory of Plateau Grazing Animal Nutrition and Feed Science Of Qinghai Province (2022-ZJ-Y17), and Top Talent project of Kunlun Talents – High-level Innovation and Entrepreneurship Talents in Qinghai Province, China (2020).

## Conflict of Interest

The authors declare that the research was conducted in the absence of any commercial or financial relationships that could be construed as a potential conflict of interest.

## Publisher's Note

All claims expressed in this article are solely those of the authors and do not necessarily represent those of their affiliated organizations, or those of the publisher, the editors and the reviewers. Any product that may be evaluated in this article, or claim that may be made by its manufacturer, is not guaranteed or endorsed by the publisher.
